# *Treponema phagedenis* in bovine digital dermatitis: biology, pathogenesis, immunity, and control strategies: a narrative review

**DOI:** 10.3389/fcimb.2026.1843191

**Published:** 2026-07-31

**Authors:** Haiyang Hu, Yixuan Wang, Yang Yang, Gang Yao, Yawei Sun, Qi Zhong, Xuelian Ma, Yuefeng Chu, Na Li

**Affiliations:** 1 Xinjiang Key Laboratory of New Drug Research and Development for Herbivorous Animals, College of Veterinary Medicine, Xinjiang Agricultural University, Urumqi, China; 2 State Key Laboratory for Animal Disease Control and Prevention, College of Veterinary Medicine, Lanzhou University, Lanzhou, China

**Keywords:** biological characteristics, bovine digital dermatitis, epidemiology, pathogenicity, *Treponema phagedenis*

## Abstract

*Treponema phagedenis* is one of the major treponemal species associated with bovine digital dermatitis (BDD), a contagious infectious hoof disease that causes lameness, reduced productivity, impaired animal welfare, and substantial economic losses in dairy and beef cattle worldwide. Since BDD was first described in 1974, the disease has been reported in many cattle-producing countries, and molecular studies have consistently identified BDD-associated *Treponema* species, including *T. phagedenis*, *T. medium*, and *T. pedis*, in active lesions. However, BDD is a polymicrobial disease, and the precise role of *T. phagedenis* as a primary pathogen, opportunistic colonizer, or contributor to lesion persistence remains incompletely resolved. This review summarizes current knowledge of the biological characteristics, global epidemiology, host range, pathogenic mechanisms, immune responses, diagnostic approaches, and prevention and control strategies related to *T. phagedenis*. Particular attention is given to culture requirements, lesion-stage progression, immune modulation, antigenic variation, candidate vaccine antigens, and recurrence after treatment. Current evidence suggests that *T. phagedenis* may contribute to chronic inflammation and lesion recurrence through tissue-associated persistence, host immune modulation, and interactions with other lesion-associated microorganisms. In China, systematic epidemiological data and confirmed local isolates remain limited, which restricts strain-level characterization and evidence-based control. Future studies should integrate culture-based isolation, molecular surveillance, comparative genomics, transcriptomics, proteomics, antimicrobial susceptibility testing, and vaccine-oriented antigen validation to clarify the role of *T. phagedenis* in BDD and improve disease prevention.

## 1 Introduction

Bovine digital dermatitis (BDD) is a contagious infectious hoof disease and one of the major causes of lameness in dairy and beef cattle worldwide. It is characterized by painful ulcerative, erosive, or proliferative lesions, most commonly located on the plantar aspect of the hind feet near the interdigital cleft and heel bulbs (Döpfer et al., 1997; Espiritu et al., 2025). Since its first description in Italy in 1974, BDD has been reported in many cattle-producing countries across Europe, North America, South America, Asia, Oceania, and Africa, and has become one of the most common and economically important infectious hoof diseases in cattle (Demirkan et al., 2006; Charfeddine and Pérez-Cabal, 2017; Relun et al., 2013b; McPherson et al., 2024; Salem et al., 2025). Affected cattle may show lameness, reduced milk yield, impaired reproductive performance, premature culling, and increased treatment costs, resulting in substantial animal welfare and economic losses (Charfeddine and Pérez-Cabal, 2017; Relun et al., 2013b). The reported prevalence of BDD varies widely among countries, herds, and production systems, partly because of differences in housing, hygiene, climate, lesion-scoring methods, sampling strategies, and diagnostic criteria (Pirkkalainen et al., 2021; Somers et al., 2005b, 2005a). In China, systematic epidemiological information remains limited, although recent studies have begun to document BDD prevalence in intensive dairy farms (Ma et al., 2024).

BDD is now widely regarded as a polymicrobial disease rather than an infection caused by a single pathogen. Anaerobic spirochetes belonging to the genus *Treponema*, particularly *T. phagedenis*, *T. medium*, and *T. pedis* or closely related phylotypes, have been consistently detected in active BDD lesions (Simon R. Clegg et al., 2016b; Döpfer et al., 1997; Evans et al., 2008; Klitgaard et al., 2013). Among these organisms, *T. phagedenis* has received increasing attention because it is frequently detected in BDD lesions, has been isolated from cattle and other host species, and may contribute to chronic lesion persistence and recurrence (Clegg et al., 2015; Simon R. Clegg et al., 2016a; Sullivan et al., 2015a, 2015b). However, whether *T. phagedenis* acts as a primary pathogen, an opportunistic colonizer, or one member of a polymicrobial pathogenic community remains unresolved. Recent genomic, transcriptomic, proteomic, and immunological studies suggest that surface-exposed proteins, antigenic variation, immune modulation, tissue invasion, and interactions with other lesion-associated microorganisms may be involved in BDD development and chronicity (Lahiri et al., 2024; Mushtaq et al., 2016; Vermeersch et al., 2022; Zuerner et al., 2007). Therefore, this narrative review summarizes current knowledge of *T. phagedenis* in relation to BDD, including its biological characteristics, epidemiology, host range, pathogenicity, immune responses, diagnostic methods, prevention strategies, and future research priorities. Because this is a narrative review, it does not provide pooled prevalence estimates or treatment effect sizes, which would require a systematic review or meta-analysis. Instead, it critically integrates recent bacteriological, genomic, transcriptomic, proteomic, immunological, diagnostic, and control-related evidence to clarify unresolved questions regarding the role of *T. phagedenis* in polymicrobial BDD lesions, with particular attention to lesion persistence, recurrence after treatment, vaccine challenges, antimicrobial resistance considerations, and research gaps in China.

## 2 Biological characteristics of *T. phagedenis*

*Treponema phagedenis* is a Gram-negative, obligately anaerobic, motile spirochete belonging to the genus *Treponema*, family *Spirochaetaceae*, and phylum *Spirochaetota* (Paster and Dewhirst, 2000; Parte et al., 2020). Cells of *Treponema* species typically exhibit slender helical morphology and periplasmic flagella, which enable corkscrew-like motility in viscous environments such as mucus, biofilms, and damaged tissue (Ruby et al., 1997; Charon et al., 2012). This motility may facilitate movement through epidermal tissue and lesion exudates, although the precise contribution of motility to BDD lesion invasion requires further experimental confirmation.

*T. phagedenis* has long been recognized as a treponemal species associated with human clinical specimens, and closely related *T. phagedenis-like* organisms have also been repeatedly detected or isolated from BDD lesions in cattle (Read and Walker, 1998; Borgmann et al., 1996; Klitgaard et al., 2008; Simon R. Clegg et al., 2016b). Molecular studies based on 16S rRNA gene sequencing, multilocus sequence typing, and comparative genomic approaches suggest that animal-associated *T. phagedenis* isolates are related to, but not necessarily identical with, human-associated lineages (Espiritu et al., 2020; Fiehn and Westergaard, 1986). This distinction is important because host-associated genetic differences may influence tissue tropism, antigenic variation, immune interaction, and pathogenic potential.

*T. phagedenis* exhibits spirochete-like motility that can be observed by dark-field microscopy. Electron microscopy has shown that cells are approximately 9–12 µm in length and 0.2–0.25 µm in diameter. The morphology of *T. phagedenis* includes a near-circular region at the midpoint of the cell and moderately helical regions at both ends (Choi et al., 1997; Wilson-Welder et al., 2013). *T. phagedenis* is a heterotrophic bacterium that utilizes primarily various carbohydrates and amino acids as carbon and energy sources, which are metabolized through fermentation. However, the utilization efficiency of *T. phagedenis* varies across different carbon sources. The bacterium grows and multiplies more rapidly when fructose, glucose, maltose, mannitol, mannose, pectin, ribose, and soluble starch serve as carbon sources. In contrast, its growth is relatively slow when arabinose, cellobiose, galactose, or lactose are used as carbon sources (Fenno, 2006; Wardle, 1997; Wilson-Welder et al., 2013). This preference may reflect its ability to adapt to specific nutrients within the host microenvironment. Furthermore, *T. phagedenis* exhibits multiple hydrolase activities, including alkaline phosphatase, C4 esterase, C8 esterase-lipase, acid phosphatase, β-galactosidase, and N-acetyl-β-D-glucosaminidase. Previous studies have shown that the growth of *Treponema* species is highly dependent on specific nutritional supplements, and that the omission of heat-inactivated fetal bovine serum (FBS), polypeptone, or yeast extract markedly impairs growth. Polypeptone and yeast extract mainly provide peptides, amino acids, vitamins, and other biosynthetic precursors. In contrast, heat-inactivated FBS likely supplies lipids, albumin-bound factors, reducing components, and protective molecules that help maintain membrane integrity and redox balance in fastidious anaerobic treponemes. Many *Treponema* species have reduced genomes and lack key pathways for *de novo* fatty acid biosynthesis as well as major antioxidant defense systems (Paster et al., 1991; Fraser et al., 1998; Norris et al., 2001). These metabolic limitations likely increase their dependence on exogenous lipids, growth factors, and reducing components supplied by FBS. Consistent with this interpretation, multiple culture studies have demonstrated that serum supplementation is required or strongly beneficial for the cultivation of *Treponema* species (Fieldsteel et al., 1981; Wardle, 1997; Fenno, 2006; Wilson-Welder et al., 2013; Arrazuria et al., 2021). Through mass spectrometry of culture supernatants, Wilson-Welder demonstrated that *T. phagedenis* produces volatile fatty acids, primarily formic acid, acetic acid, and butyric acid (Wilson-Welder et al., 2013). These metabolites may represent end products of its fermentative metabolism or potentially play roles in host–microbe interactions.

The outer membrane of *T. phagedenis* contains proteins and lipoproteins that may be involved in adhesion, host interaction, immune recognition, and antigenic variation. Proteomic and immunological studies have identified several immunogenic or surface-associated proteins, including PrrA, TmpA, Ttm, VpsA, VpsB, and putative β-barrel outer membrane proteins, as potential contributors to host-pathogen interaction or vaccine development (Mushtaq et al., 2016; Staton et al., 2020; Lahiri et al., 2024). However, the biological functions and protective relevance of many of these proteins remain incompletely defined.

Overall, the biological characteristics of *T. phagedenis* suggest that it is well adapted to anaerobic, nutrient-rich, and damaged epithelial environments. Its motility, fastidious growth requirements, surface antigen diversity, and possible immune-interactive proteins may contribute to persistence in BDD lesions. Nevertheless, these traits should be interpreted in the context of BDD as a polymicrobial disease, because lesion development likely depends on interactions among *T. phagedenis*, other BDD-associated treponemes, anaerobic bacteria, the local tissue environment, and host immune responses (Watts et al., 2018; Klitgaard et al., 2013; Zinicola et al., 2015b).

## 3 Global epidemiology and detection of BDD-associated *Treponema* species

**Figure 1:**
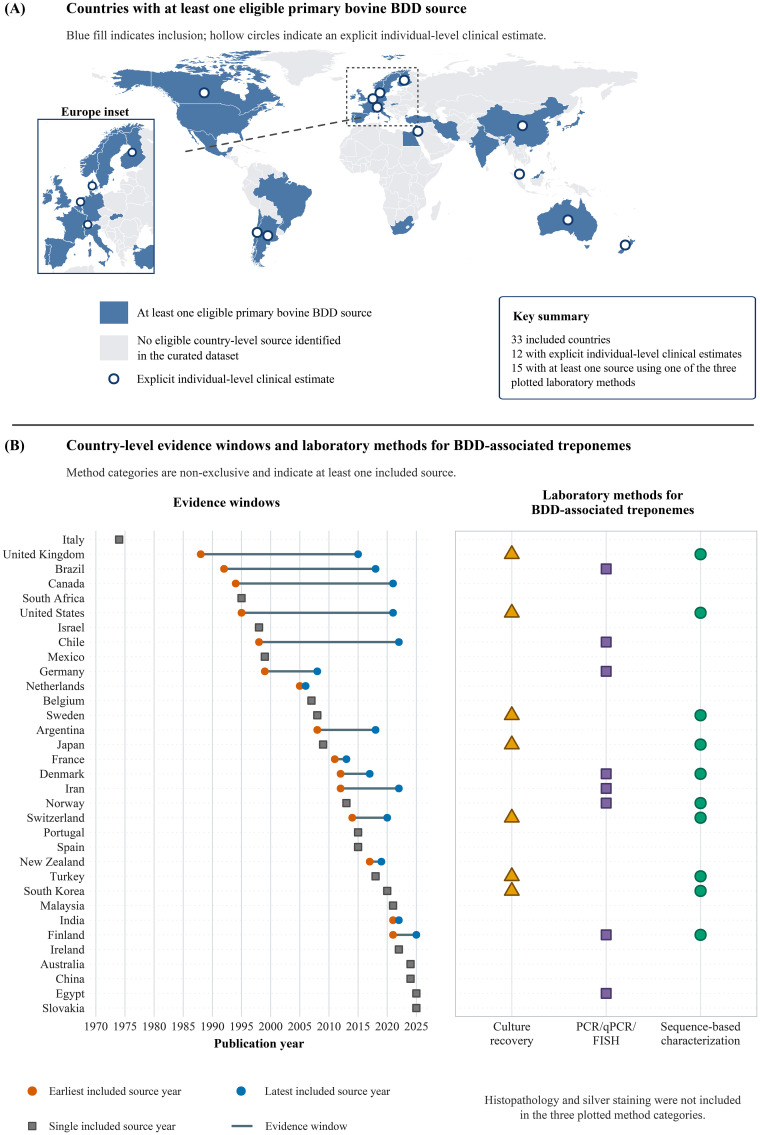
Global reported distribution of bovine digital dermatitis and country-level evidence synthesis as of 31 December 2025. **(A)** Countries for which at least one eligible primary bovine digital dermatitis (BDD) source was identified in the curated dataset. Blue fill indicates inclusion, whereas gray indicates that no eligible country-level source was identified in the curated dataset; gray should not be interpreted as evidence that BDD is absent or that no BDD report exists for that country. Hollow circles identify countries with at least one explicit individual-level clinical estimate. An estimate was classified as individual-level when a primary source reported a clinical BDD proportion or percentage for a defined bovine study population. Herd-level positivity, hoof- or lesion-level proportions, combined lesion outcomes, lameness, serology, and pathogen-detection proportions were excluded. Because sampling frames, case definitions, and ascertainment procedures differed among studies, the markers indicate the availability of an estimate rather than nationally representative prevalence or direct cross-country comparability. **(B)** Country-level evidence windows and laboratory methods for BDD-associated treponemes across the 33 included countries. In the left subpanel, orange and blue circles denote the earliest and latest years among the included sources, respectively; gray squares denote a single included-source year, and horizontal segments connect the earliest and latest included years. These segments are bibliographic evidence windows and do not imply continuous surveillance, uninterrupted disease occurrence, national incidence, or endemicity. In the right subpanel, symbols indicate whether at least one included source reported culture recovery, targeted detection by PCR, qPCR, or FISH, or sequence-based characterization of BDD-associated treponemes. Sequence-based characterization includes sequencing, typing, phylogenetic analysis, or taxonomic assessment. Method categories are non-exclusive, and the absence of a symbol means only that the corresponding method was not identified in the included source set. Culture recovery does not by itself establish formal species-level assignment. Histopathology and silver staining were not included in the three plotted method categories. Literature cutoff: 31 December 2025.

Since its first report in 1974, BDD has been confirmed in numerous cattle-producing countries worldwide (Demirkan et al., 2006). **Figure 1** provides a descriptive synthesis of eligible country-level bovine BDD sources, explicit individual-level clinical estimates, bibliographic evidence windows, and laboratory methods reported for BDD-associated treponemes; these indicators reflect the availability of published evidence rather than nationally representative prevalence, continuous surveillance, or endemicity. Although no single global core epidemic area has been clearly defined, BDD has become one of the most common infectious hoof diseases causing lameness in cattle. *T. phagedenis* and closely related BDD-associated treponemes are most frequently reported in cattle, although similar organisms have also been detected in other hoofed animals. Infection rates and lesion severity may vary among herds and production systems, and may be influenced by breed, age, housing, hygiene, climate, and management practices. Bernhard reported the presence of spirochetes in sheep foot lesions (Bernhard et al., 2021). Within flocks with a history of lameness, Calvo-Bado detected spirochetes in sheep with pododermatitis lesions (Calvo-Bado et al., 2011), whereas Frosth identified spirochetes in the feet of both lame and healthy sheep flocks (Frosth et al., 2012). Paraschou reported that spirochetes associated with BDD pathogenesis were isolated from 30% of the 167 donkey horn tumor samples in the total sample population (Paraschou et al., 2023). Hoby also detected the presence of *T. phagedenis* in hoof dermatitis cases among captive European bison populations at Bern Zoo (Hoby et al., 2021). Clegg detected and isolated *Treponema* spp. from the affected sites of moose hooves but not from other unaffected hoof areas or control hooves (Clegg et al., 2015). Han reported that up to 80% of wild moose herds in the United States comprised lame moose, with 30% to 90% of individuals in affected geographic areas suffering from hoof disease (Han et al., 2019). Reported animal hosts and associated detection methods are summarized in **Table 1**.

**Table 1:** Reported animal hosts and human-associated isolates of *T. phagedenis* and BDD-associated treponemes.

Host species	Disease or lesion type	Detected organism or group	Detection method	Region	Reference
Cattle	Bovine digital dermatitis	*T. phagedenis*, *T. medium*, *T. pedis*	Culture, PCR, sequencing, FISH	Multiple countries	Simon R. Clegg et al., 2016b; Döpfer et al., 1997; Klitgaard et al., 2013
Sheep	Contagious ovine digital dermatitis or foot lesions	BDD-associated treponemes	PCR, sequencing	Sweden/UK and other regions	Bernhard et al., 2021; Calvo-Bado et al., 2011; Frosth et al., 2012
Donkey	Keratoma or horn lesions	*Treponema* spp.	Culture, sequencing	UK	Paraschou et al., 2023
European bison	Digital dermatitis lesions	*T. phagedenis* and related treponemes	PCR, histology	Switzerland	Hoby et al., 2021
Wild elk/moose	Hoof disease or DD-like lesions	BDD-associated treponemes	Culture, PCR, sequencing	North America	Clegg et al., 2015; Han et al., 2019
Human	CSF from neurosyphilis patient	*T. phagedenis*	mNGS, phylogenetic analysis	China	Yuan et al., 2023^1

^1Yuan, C., et al. (2023). Case Report: Detection of *Treponema phagedenis* in cerebrospinal fluid of a neurosyphilis patient by metagenomic next-generation sequencing. Front. Cell. Infect. Microbiol. 13:1218049. doi: 10.3389/fcimb.2023.1218049.

This is the first documented case of *T. phagedenis* detection in human CSF in China; phylogenomic analysis showed it belongs to a human-associated lineage distinct from BDD-associated bovine strains.

Although these findings suggest that BDD-associated treponemes can be detected in multiple hoofed species, the evidence does not yet prove frequent cross-species transmission. Treponemes detected in cattle and sheep lesions appear closely related in several molecular epidemiological studies, whereas treponemes from other host species may belong to different phylotypes or host-associated lineages (Sullivan et al., 2015b; Simon R. Clegg et al., 2016b; Hoby et al., 2021; Paraschou et al., 2023). Therefore, host range should be interpreted cautiously, and current evidence is insufficient to conclude that there is a high risk of broad cross-species transmission. Future studies should combine strain-level genotyping, comparative genomics, and epidemiological tracing to determine whether isolates from different hosts represent shared transmission chains or host-adapted lineages.

The incidence of BDD may exhibit seasonal peaks (such as during humid seasons), but specific patterns depend on the local climate and management practices. The geographical extent of the published BDD evidence base has expanded over time. However, this increase in reporting should not be interpreted as evidence of a global rise in prevalence or incidence, because surveillance intensity, study design, diagnostic criteria, and reporting practices differ among countries and periods. BDD spreads primarily through direct contact (between infected and healthy cattle) and environmental contamination. Instruments such as hoof trimmers serve as significant mechanical vectors. Research has indicated that *T. phagedenis* can survive on hoof trimmers for up to 2 hours under aerobic conditions, suggesting that inadequate disinfection of these tools poses a risk for transmission between farms (Gillespie et al., 2020). Factors that significantly increase infection and disease risk include environmental and management factors such as wet, muddy ground, poor hoof hygiene, overcrowding, hoof injuries, lax management, and stress, as well as potential nutritional imbalances (e.g., zinc deficiency) and concurrent infections.

Cultures from BDD lesions have yielded numerous bacterial species, including *Clostridium* spp., *Bacteroides* spp., *Campylobacter* spp., and *Peptostreptococcus* spp (Ohya et al., 1999). According to histopathological examination, spirochetes are most commonly detected in the epidermis and deep dermis of affected skin tissues in BDD (Ce et al., 2005; Read and Walker, 1998). Further studies on the genetic and antigenic diversity of the pathogen revealed the presence of multiple spirochete species in BDD lesions; however, very few spirochetes have been successfully cultured from BDD lesions (Klitgaard et al., 2013). In 1995, Walker et al. reported the successful isolation and culture of spirochetes from cattle with BDD in the United States (Walker et al., 1995). In 1999, *Treponema* spp. were first recovered from BDD lesions in the UK using immunomagnetic separation (Demirkan et al., 1999). Independently, Schrank et al. described a novel species, *Treponema brennaborense*, from bovine pododermatitis in Germany (Schrank et al., 1999). Subsequent molecular phylogenetic analyses identified three distinct *Treponema* phylotypes consistently associated with BDD — *Treponema medium*, *T. pedis*, and *T. phagedenis* — thereby establishing the polymicrobial etiology of this disease (Clegg et al., 2016b; Döpfer et al., 2012a).

## 4 Pathogenicity of *Treponema phagedenis* in bovine digital dermatitis

BDD is generally considered a polymicrobial disease rather than an infection caused by a single bacterial species. High-throughput sequencing, fluorescence *in situ* hybridization, and culture-based studies have consistently shown that active BDD lesions contain complex bacterial communities dominated by anaerobic taxa, including several BDD-associated *Treponema* phylotypes such as *T. phagedenis*, *T. medium*, and *T. pedis* (Arrazuria et al., 2020; Klitgaard et al., 2013, 2008). Therefore, the pathogenic role of *T. phagedenis* should be interpreted as part of a polymicrobial lesion environment, where interactions among treponemes, other anaerobic bacteria, damaged epidermal tissue, and the local immune response may jointly contribute to lesion development and persistence.

The development of BDD lesions is commonly described using lesion-stage classification systems, including early-stage, active ulcerative, healing, chronic, and recurrent lesions. The principal clinical features of the M0–M4.1 stages are summarized in **Table 2**. These clinical stages reflect dynamic changes in lesion morphology, bacterial composition, and host tissue responses (Döpfer et al., 1997; Zinicola et al., 2015b). Active lesions are often painful, moist, ulcerative, and highly inflammatory, whereas chronic lesions may become hyperkeratotic, proliferative, or recurrent. The frequent recurrence of BDD after apparent clinical improvement suggests that lesion resolution is often incomplete and that treponemes or other lesion-associated bacteria may persist in the local tissue or farm environment (Relun et al., 2013b; Zinicola et al., 2015b).

**Table 2:** Characteristics of symptoms at different stages of BDD.

M stage	Stage of the disease	Clinical manifestations
M0	Healthy Skin	Hoof skin is intact with no discoloration, and palpation reveals no pain response.
M1	Early stage	Hoof skin infection caused by one or more pathogenic microorganisms, resulting in small localized lesions gradually developing on the skin. The affected area measures less than 2 cm in diameter and presents as a gray or red, rough ulcerated surface. When pressure is applied to the lesion, the cow exhibits a pain response.
M2	Acute phase	The infection is intensified, with the lesion gradually expanding to at least 2 cm in diameter, accompanied by symptoms such as ulceration and fissures between the toes.
M3	Healing stage	The affected area gradually forms a scab and hardens, turning brown. Pressing firmly on the lesion elicits no response or pain.
M4	Chronic stage	The skin between the toes becomes hardened and develops grayish-white or brown filamentous and crust-like growths, which are painless upon pressure.
M4.1	Chronic-active stage	Transitional stage with small (< 0.5 cm) ulcerative changes within a chronic M4 lesion containing a small active M1 focus, preceding relapse to the acute M2 stage; reflects inadequate hoof management during the chronic phase.

Adapted from Döpfer et al., 1994; Döpfer et al., 1997.

In addition to helical motile forms, BDD-associated treponemes may undergo morphological changes under stressful or chronic lesion conditions. Round-body-like or altered morphological forms have been described in treponemal cultures and may reflect adaptation to unfavorable environments, although their biological significance *in vivo* remains uncertain (Döpfer et al., 2012a; Wilson-Welder et al., 2013). If such forms occur in chronic BDD lesions, they could potentially contribute to persistence or recurrence by reducing metabolic activity or altering exposure to antimicrobial agents and host immune factors. However, direct evidence linking round-body formation of *T. phagedenis* to BDD recurrence remains limited and requires further microscopic, culture-based, and molecular validation.

Several biological features of *T. phagedenis* may help explain its association with active and chronic BDD lesions. As a motile spirochete, *T. phagedenis* has the potential to move through viscous lesion exudates and damaged epithelial tissue, a property that is characteristic of spirochetes with periplasmic flagella (Charon et al., 2012). In addition, BDD-associated treponemes have been detected deep within the epidermis and near the dermal-epidermal junction in lesion tissues, supporting the view that treponemes are not merely superficial contaminants but may invade or persist within damaged skin structures (Klitgaard et al., 2013, 2008).

Experimental and transcriptomic studies further support the view that BDD-associated treponemes differ in pathogenic potential and may activate distinct host inflammatory pathways. Newbrook et al. demonstrated that *T. phagedenis* elicits proinflammatory responses involving IL-8 and IL-17 signaling in bovine digital dermal fibroblasts (Newbrook et al., 2020). In a longitudinal transcriptomic study of acute and chronic BDD lesions, Vermeersch et al. identified a shared core transcriptional signature characterized by upregulation of inflammatory genes such as A2ML1, PI3, and CCL11, together with downregulation of keratin genes and anti-inflammatory mediators including SCGB1D (Vermeersch et al., 2022). Pathway analysis further revealed persistent activation of the IL-17F-driven inflammatory pathway in both acute and chronic lesions, indicating that BDD pathology is associated with excessive inflammatory activation, impaired epithelial barrier integrity, and delayed wound repair. Critically, substantial inflammatory activity remained detectable in M3 and M4 lesions despite apparent clinical healing. The pronounced rebound of proinflammatory cytokine expression during the M4.1 phase, coinciding with the emergence of acute lesions, suggests that clinically quiescent lesions may retain an inflammatory and microbiologically permissive state before relapse (Vermeersch et al., 2022). This finding supports the concept that chronic M4 lesions can act as reservoirs for recurrence, although direct evidence for latent persistence of *T. phagedenis* within tissues remains limited. Such subclinical lesion activity may be missed during routine hoof trimming and visual inspection, thereby sustaining herd-level transmission if chronic and recurrent lesions are not actively monitored (Relun et al., 2013a; Zinicola et al., 2015a).

Distinct Treponema species associated with BDD may activate different host-response pathways. Transcriptomic analyses revealed that *T. phagedenis* and *T. pedis* are associated with upregulation of apoptosis-related transcripts, whereas *T. medium* and *T. pedis* are associated with genes involved in actin cytoskeletal remodeling and loss of cell adhesion, processes that may contribute to tissue disruption or invasion (Newbrook et al., 2020). Notably, elevated expression of the antimicrobial peptide precursor DEFB123 in response to *T. phagedenis* may reflect a distinct host response within the polymicrobial BDD lesion environment. Host transcriptomic responses induced by BDD-associated treponemes have been compared with responses to *T. pallidum*, suggesting that these organisms may share some immune-interactive features with other pathogenic treponemes. However, direct evidence for defined immune evasion mechanisms in BDD-associated *Treponema* species remains limited. Collectively, these findings support the possibility that *T. phagedenis* contributes to persistence and recurrence in chronic BDD lesions, but they do not prove that *T. phagedenis* alone is sufficient to initiate or maintain BDD.

## 5 Immune responses to *T. phagedenis* and BDD-associated treponemes

The host immune response to BDD is characterized by local inflammation, epithelial barrier disruption, and incomplete bacterial clearance. Active BDD lesions are typically painful, ulcerative, and inflamed, suggesting strong activation of local innate immune responses (Berry et al., 2012; Dörte Döpfer et al., 2012b). Molecular studies have shown that BDD lesions contain abundant treponemes and other anaerobic bacteria, which may provide persistent microbial stimulation to keratinocytes, fibroblasts, and resident immune cells (Klitgaard et al., 2013, 2008).

Serological studies have provided evidence that BDD-associated treponemes elicit measurable host antibody responses. Demirkan et al. first demonstrated antispirochete antibody responses in BDD-affected cattle that were absent in healthy animals, suggesting seroconversion associated with infection (Demirkan et al., 1998). Subsequently, Moe et al. measured serum IgG levels against three *T. phagedenis* strains by ELISA in 85 BDD-positive and 33 control cattle and reported significantly higher anti-*T. phagedenis* IgG titers in BDD-positive animals (Moe et al., 2010). These findings indicate that cattle with BDD can mount a measurable humoral response against *T. phagedenis* antigens, although the presence of antibodies alone does not necessarily indicate protective immunity.

Macrophages are central mediators of the innate immune response to *Treponema* infections. Early histopathological studies in experimental rabbit syphilis first established their role in controlling *T. pallidum*, and subsequent *in vitro* work demonstrated efficient phagocytic clearance by peritoneal macrophages (Lin et al., 2018). Structurally conserved components of *Treponema* spp. stimulate secretion of CXCL-8, IL-10, IL-6, IL-12, IL-1β, and TNF-α in periodontal cells (Refaai et al., 2013; Sellati et al., 1998). In the BDD context, Lahiri et al. demonstrated that *T. phagedenis* activates bovine macrophages through NLRP3 inflammasome-dependent signaling, triggering IL-1β release. Compared with healthy skin, BDD lesions harbored elevated CD14-high and CD16-low monocyte/macrophage populations, and this phenotype persisted despite antibiotic treatment (Lahiri et al., 2024). Although *T. phagedenis* and *T. pallidum* share phylogenetic affinity, they differ substantially in host range, disease manifestation, and tissue tropism. Whether their pathogenic and immune evasion strategies are conserved remains an open question lacking sufficient experimental support.

Moe et al. measured serum IgG levels against three *T. phagedenis* strains by ELISA using 85 BDD-positive and 33 control samples. BDD-positive animals exhibited significantly higher anti-*T. phagedenis* IgG titers than controls, confirming that *T. phagedenis* infection elicits a measurable humoral immune responses (Moe et al., 2010). More recent work has further examined immune responses to digital dermatitis pathogen-derived antigens during infection, recovery, and reinfection. Coatney et al. reported that immune responses to BDD-associated antigens vary across disease stages and may not provide complete protection against reinfection, highlighting the complexity of protective immunity in BDD (Coatney et al., 2024). These findings are consistent with the frequent recurrence of BDD after apparent clinical improvement and suggest that measurable humoral or cellular responses do not necessarily equate to durable protective immunity.

Antigenic variation represents a key immune evasion strategy of bacterial pathogens, occurring through gene rearrangements that alter surface protein expression and regulatory switches between distinct expression states (Vink et al., 2012). Mushtaq et al. identified two immunogenic lipoproteins, VpsA and VpsB, encoded by genes located in close proximity to the previously characterized antigenic protein PrrA on the *T. phagedenis* chromosome (Mushtaq et al., 2016). Comparative analysis of this locus across 15 *T. phagedenis* isolates revealed allelic variants of *vpsA* and *prrA*, along with variable (TA)n repeat numbers within the putative promoters that may regulate phase and antigenic variation of these surface antigens, providing a plausible molecular basis for antigenic variation and potential immune evasion by *T. phagedenis.*

Several immunogenic or surface-associated proteins of *T. phagedenis*, including PrrA, VpsA, VpsB, TmpA, Ttm, and putative β-barrel outer membrane proteins, have been proposed as potential vaccine antigens (Lahiri et al., 2024; Mushtaq et al., 2016; Staton et al., 2020). These proteins may participate in host interaction, immune recognition, or antigenic variation and therefore represent rational targets for vaccine-oriented studies. However, vaccine availability for BDD remains limited, and no vaccine has been widely validated as consistently effective for field prevention of BDD-associated treponemal infection. Vaccine development is hindered by the polymicrobial etiology of BDD, antigenic diversity among *Treponema* isolates, incomplete understanding of protective immune correlates, limited strain-level surveillance, the fastidious anaerobic nature of *T. phagedenis*, and the lack of stable and reproducible challenge models (S. R. Clegg et al., 2016a; Coatney et al., 2024; Mushtaq et al., 2016). Future vaccine research should combine comparative genomics, surface proteomics, immunological screening, and controlled animal studies to identify conserved protective antigens.

## 6 Diagnosis and prevention of *T. phagedenis*

BDD diagnosis currently relies primarily on clinical lesion staging using the M-score system under field conditions (Döpfer et al., 1997). Morphological identification of spirochetes in lesion tissues can be achieved by dark-field or phase-contrast microscopy, though sensitivity is limited (Sayers et al., 2009; Edmondson et al., 2025). Molecular approaches, including PCR, nested PCR, and quantitative PCR, detect *Treponema* DNA in lesion samples with substantially greater sensitivity and specificity and can identify multiple BDD-associated treponemal phylotypes or species (Klitgaard et al., 2013; Frosth et al., 2023). Serological assays, including ELISA, detect host antibody responses against *Treponema* antigens (Afonso et al., 2021; Roelofs et al., 2024). Fluorescence *in situ* hybridization (FISH) is widely used as a reference method for visualizing and localizing BDD-associated treponemes directly within lesion tissues. Compared with PCR, which detects bacterial DNA but does not show spatial distribution, FISH can demonstrate whether treponemes are present within the epidermis, dermal-epidermal junction, or deeper lesion structures, thereby providing strong evidence of tissue association rather than surface contamination (Klitgaard et al., 2013, 2008). Therefore, combining clinical M-stage scoring, PCR/qPCR, and FISH provides a more reliable diagnostic framework for research studies than clinical inspection alone. Moreover, the difficulty of cultivating *T. phagedenis* from BDD lesions has limited the application of culture-based diagnosis, representing a primary diagnostic bottleneck (Klitgaard et al., 2013).

Antimicrobial and topical treatments remain important for managing active BDD lesions, especially painful M2 lesions. Topical oxytetracycline has been widely used for individual lesion treatment and has shown short-term clinical efficacy in reducing lesion severity and promoting healing, although recurrence after treatment remains common (Laven and Logue, 2006; Relun et al., 2013a). Other topical products, including salicylic acid paste, non-antibiotic gels, and wound dressings, have also been evaluated as alternatives or adjuncts to antibiotic therapy. Weber et al. compared salicylic acid paste and polyurethane wound dressings in German Holstein dairy cows with BDD and reported comparable therapeutic efficacy between the two treatments (Weber et al., 2019). These findings suggest that non-antibiotic approaches may be useful in reducing reliance on antimicrobial agents, particularly in herds with frequent recurrent lesions.

Because BDD is associated with anaerobic treponemes and polymicrobial lesion communities, disinfectants and topical antiseptics have also been investigated. Gillespie et al. showed by phase-contrast microscopy that 1% FAM30, 2% Virkon, and 2% sodium hypochlorite completely inhibited visible growth of BDD-associated treponemes under laboratory conditions (Gillespie et al., 2020). However, *in vitro* inhibition does not necessarily translate directly into field-level efficacy, because organic matter, lesion depth, biofilm-like microbial organization, and inadequate contact time may reduce disinfectant performance. Therefore, antimicrobial and antiseptic treatment should be combined with lesion cleaning, appropriate contact time, and herd-level prevention measures.

Antimicrobial susceptibility testing of BDD-associated treponemes remains limited because *T. phagedenis* and related treponemes are fastidious anaerobes and are difficult to isolate as pure cultures from field lesions. Nevertheless, several *in vitro* MIC studies have provided useful preliminary evidence. Evans et al. tested bovine digital dermatitis-associated spirochetes and reported that many isolates were susceptible to commonly used agents such as penicillin, ampicillin, erythromycin, and tetracycline, although susceptibility varied among isolates and antimicrobial classes (Evans et al., 2009). Yano et al. further evaluated *T. phagedenis*-like spirochetes isolated from dairy cattle with papillomatous digital dermatitis in Japan and showed low MIC values for several β-lactams, macrolides, and tetracycline-class drugs, supporting their *in vitro* activity against treponemal isolates (Yano et al., 2010). More recently, genotypic and phenotypic characterization of *T. phagedenis* from BDD also indicated that ampicillin, erythromycin, and tetracycline could inhibit the growth of tested isolates at relatively low concentrations (Arrazuria et al., 2020). However, these results should be interpreted cautiously because *in vitro* susceptibility does not necessarily predict clinical cure in BDD lesions, where biofilm-like microbial communities, necrotic tissue, lesion depth, organic matter, and poor topical contact may reduce treatment efficacy.

In addition to MIC-based studies, antimicrobial resistance gene analyses have raised concerns that BDD lesions may act as reservoirs of resistance determinants. Metagenomic and metagenomic Hi-C analyses have identified multiple antimicrobial resistance genes in BDD-associated microbial communities, including tetracycline resistance genes such as *tetQ*, *tetM*, and other efflux or ribosomal protection-associated determinants, which were often linked to non-treponemal anaerobes within the polymicrobial lesion community rather than exclusively to *Treponema* spp (Beyi et al., 2021; Zinicola et al., 2015a). This suggests that repeated topical antibiotic use may not only affect *T. phagedenis*, *T. medium*, and *T. pedis*, but may also select for resistant bacteria in the broader lesion microbiome. Therefore, susceptibility monitoring should combine culture-based MIC testing of BDD-associated treponemes with molecular surveillance of resistance genes in whole lesion communities. Future studies should establish standardized anaerobic culture, MIC testing, genome sequencing, and resistome analysis protocols for *T. phagedenis*, *T. medium*, and *T. pedis*, especially for local isolates from China, to support evidence-based treatment recommendations and reduce unnecessary antimicrobial use.

Recent practical reviews emphasize that effective BDD control requires integrated herd-level programs rather than reliance on treatment of individual lesions alone. Such programs should combine early detection, prompt treatment of active lesions, regular foot disinfection, improved hygiene, biosecurity, and monitoring of chronic lesions (Bell and Vanhoudt, 2020). This is particularly important because chronic M4 and recurrent M4.1 lesions can maintain infection pressure within the herd even when acute lesions are treated. Regular medicated footbaths represent one of the most widely used preventive measures in dairy herds. Footbaths can reduce pathogen load on the hoof surface and lower the risk of new infections when properly designed, maintained, and refreshed (Jacobs et al., 2019). Commonly used footbath agents include copper sulfate, formaldehyde, glutaraldehyde, and potassium persulfate compounds, which have disinfectant or antiseptic properties (Brennan and Stavisky, 2020; Ferraro et al., 2024). However, their effectiveness depends on concentration, contamination by manure, frequency of use, cow flow, lesion prevalence, and farm hygiene. Therefore, footbath protocols should be adapted to herd conditions and evaluated using standardized lesion scoring.

Hoof trimming is another important component of BDD prevention and control. Preventive hoof trimming helps maintain hoof balance, removes overgrown or damaged horn tissue, improves weight distribution, and reduces local trauma that may predispose cattle to infection (Pedersen et al., 2022). However, trimming should be performed carefully, because excessive or improper trimming may damage hoof tissue and increase infection risk. Hoof knives and trimming equipment may also act as mechanical vectors for BDD-associated treponemes. Gillespie et al. reported that BDD treponemes can survive on hoof knife blades for up to 2 h under aerobic conditions, emphasizing the need for strict cleaning and disinfection between animals and farms (Gillespie et al., 2020).

Nutritional and environmental management also contribute to BDD prevention. Wet floors, poor hygiene, high stocking density, abrasive or poorly drained walking surfaces, and accumulation of manure increase exposure of the interdigital skin to anaerobic microorganisms and may compromise skin barrier integrity (Cramer et al., 2018; Holzhauer et al., 2006; Relun et al., 2013a; Somers et al., 2005b, 2005a). Improving walking surfaces, drainage, bedding cleanliness, stocking density, and hoof hygiene can therefore reduce environmental contamination and transmission pressure within the herd (Jacobs et al., 2019; Pedersen et al., 2022). Zinc supplementation may support hoof horn quality, epithelial integrity, and wound repair. Wenner et al. reported that zinc source influenced the relative abundance of fecal *Treponema* spp. in lactating dairy cows, suggesting that mineral nutrition may affect treponemal ecology, although direct evidence for zinc supplementation preventing BDD remains limited (Wenner et al., 2022). Therefore, zinc should be considered a supportive nutritional strategy rather than a stand-alone preventive measure.

Recent field-oriented studies have also emphasized the importance of farm-specific risk assessment and implementation quality. Gillespie et al. evaluated key practices for controlling digital dermatitis and highlighted that solution depth, footbath hygiene, animal flow, and the potential of contaminated footbaths to act as reservoirs for Treponema species should be considered when designing control programs (Gillespie et al., 2024). Similarly, Weber et al. described successful implementation of a herd-level risk assessment and mitigation program, supporting the view that BDD control requires continuous monitoring, identification of farm-specific risk factors, and iterative adjustment of preventive measures rather than occasional treatment of visible lesions alone (Weber et al., 2025).

From a practical perspective, BDD control should combine early lesion detection, standardized M-stage recording, prompt treatment of active lesions, regular hoof hygiene, effective footbath management, disinfection of hoof-trimming equipment, improvement of housing conditions, and monitoring of chronic M4 and recurrent M4.1 lesions. Because BDD recurrence is common, herd programs should focus not only on healing visible lesions but also on reducing environmental contamination and identifying cows with chronic or recurrent lesions that may serve as reservoirs for transmission.

## 7 Conclusion and future perspectives

In conclusion, *T. phagedenis* is one of the major treponemal species associated with BDD, but it should not be regarded as the sole causative agent of the disease. Its precise role as a primary pathogen, opportunistic colonizer, or persistence-associated member of a polymicrobial lesion community remains unresolved. Current evidence suggests that its fastidious anaerobic growth, tissue-associated persistence, surface antigen diversity, immune modulation, and interactions with other lesion-associated microorganisms may contribute to chronic inflammation and recurrence. However, the pathogenic mechanisms of *T. phagedenis* should be interpreted together with other BDD-associated treponemes, especially *T. medium* and *T. pedis*, and with anaerobic bacteria in the lesion microbiome.

Major gaps remain in culture-based isolation, comparative genomics, antimicrobial susceptibility testing, vaccine antigen validation, and field-level prevention strategies. In China, systematic epidemiological data and confirmed local isolates remain particularly limited, restricting strain-level characterization and evidence-based control. Future studies should integrate molecular surveillance, culture-based isolation, host-response analysis, comparative genomics, transcriptomics, proteomics, antimicrobial susceptibility testing, and vaccine-oriented immunological validation to clarify the biological role of *T. phagedenis* and improve BDD prevention and control.
